# Data-driven multiscale modeling reveals the role of metabolic coupling for the spatio-temporal growth dynamics of yeast colonies

**DOI:** 10.1186/s12860-019-0234-z

**Published:** 2019-12-19

**Authors:** Jukka Intosalmi, Adrian C. Scott, Michelle Hays, Nicholas Flann, Olli Yli-Harja, Harri Lähdesmäki, Aimée M. Dudley, Alexander Skupin

**Affiliations:** 1 Department of Computer Science, Aalto University, P.O.Box 15400, Aalto, FI-00076 Finland; 2 Pacific Northwest Research Institute, 720 Broadway, Seattle, WA, 98122 USA; 3 Molecular and Cellular Biology Program, University of Washington, Seattle, WA, 98195 USA; 4 Department of Computer Science, Utah State University, 4205 Old Main Hill, Logan, UT, 84322 USA; 5 BioMediTech and Faculty of Biomedical Sciences and Engineering, Tampere University of Technology, P.O.Box 553, Tampere, 33101 Finland; 6 Institute for Systems Biology, 1441N 34th Street, Seattle, WA, 98103-8904 USA; 7 Luxembourg Centre for Systems Biomedicine, University of Luxembourg, 2, avenue de l’Université, Esch-sur-Alzette, L-4365 Luxembourg; 8 University of California San Diego, 9500 Gilman Dr, La Jolla, CA, 92093 USA

**Keywords:** Multicellular systems, Multiscale modeling, Yeast colony, Metabolic coupling, Diauxic shift, Markov chain Monte Carlo, Bayesian optimization

## Abstract

**Background:**

Multicellular entities like mammalian tissues or microbial biofilms typically exhibit complex spatial arrangements that are adapted to their specific functions or environments. These structures result from intercellular signaling as well as from the interaction with the environment that allow cells of the same genotype to differentiate into well-organized communities of diversified cells. Despite its importance, our understanding how this cell–cell and metabolic coupling lead to functionally optimized structures is still limited.

**Results:**

Here, we present a data-driven spatial framework to computationally investigate the development of yeast colonies as such a multicellular structure in dependence on metabolic capacity. For this purpose, we first developed and parameterized a dynamic cell state and growth model for yeast based on on experimental data from homogeneous liquid media conditions. The inferred model is subsequently used in a spatially coarse-grained model for colony development to investigate the effect of metabolic coupling by calibrating spatial parameters from experimental time-course data of colony growth using state-of-the-art statistical techniques for model uncertainty and parameter estimations. The model is finally validated by independent experimental data of an alternative yeast strain with distinct metabolic characteristics and illustrates the impact of metabolic coupling for structure formation.

**Conclusions:**

We introduce a novel model for yeast colony formation, present a statistical methodology for model calibration in a data-driven manner, and demonstrate how the established model can be used to generate predictions across scales by validation against independent measurements of genetically distinct yeast strains.

## Background

Multicellular organisms and colonies of unicellular microbes are able to form characteristic structures by specialized cell types [1, 2]. While it is generally accepted that the structure and functions of tissue and organs are genetically encoded, more recently it has been demonstrated that the morphologies of biofilms like *Saccharomyces cerevisiae* yeast colonies have a strong genetic component [3–5]. Together with the frequently observed growth medium dependency of yeast cultures, these findings further underpin the importance of genome–environment interactions for phenotype development [6]. To understand the underlying mechanisms, it is key to investigate how metabolic coupling is influencing individual cell states and instructing structure formation.

**Fig. 1:**
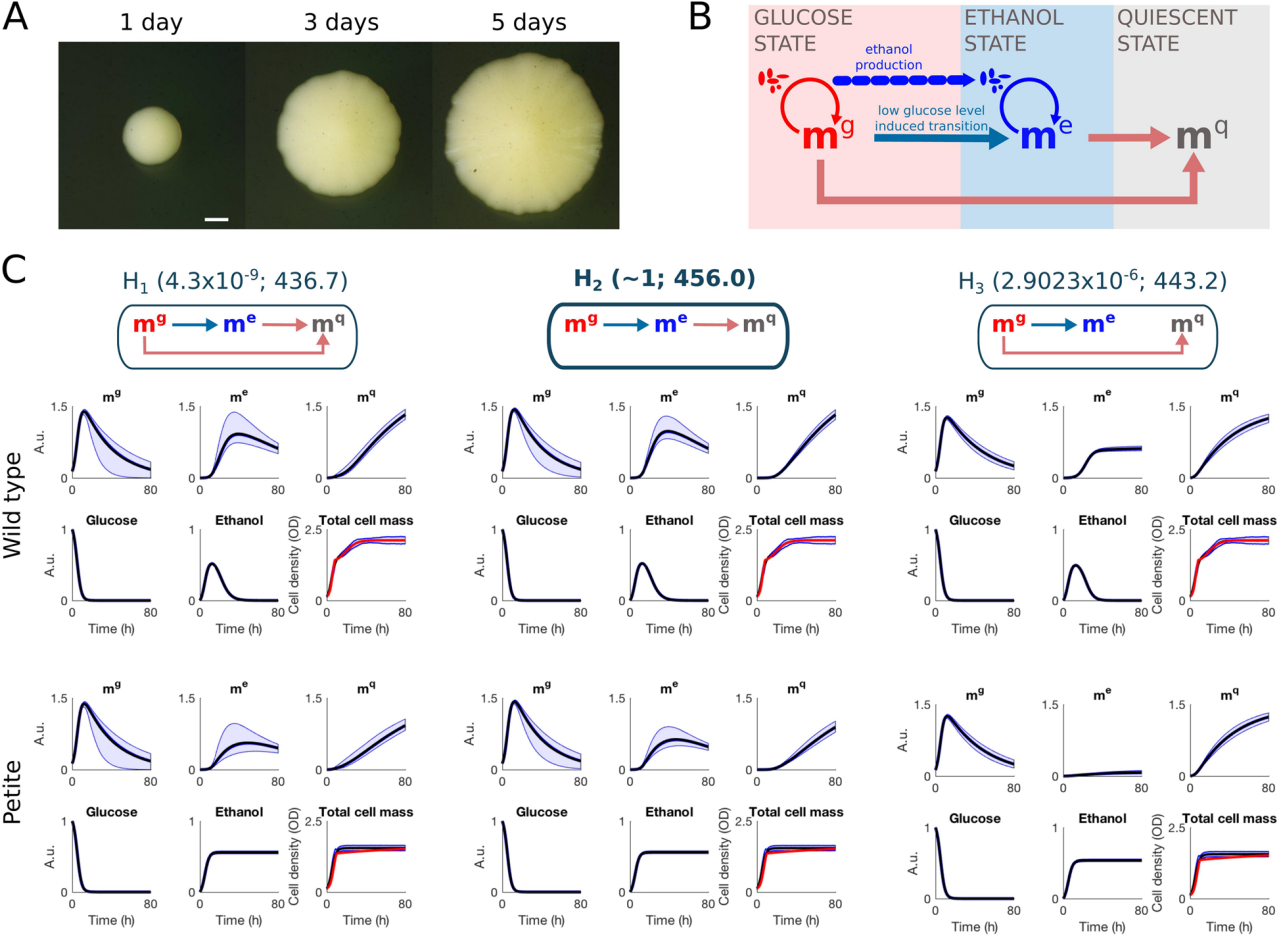
Illustration of real colony growth and summary of microenvironment model inference. **a** A real colony growing on a nutrient rich agar. **b** Schematic illustration of the microenvironment model. **c** Illustration of the alternative metabolic switching routes (hypotheses *H*_1_,*H*_2_, and *H*_3_) and summary of microenvironment model inference. The hypothesis *H*_1_ contains both possible transitions from the glucose state to the quiescent state and the hypotheses *H*_2_ and *H*_3_ can be obtained by removing one of the routes (these hypotheses correspond to setting the switching rate parameters *β*_2_ and *β*_3_ in the model to zero, respectively). Each hypothesis is accompanied with the posterior probability and the estimated logarithmic marginal likelihood (shown in parentheses after hypothesis). The estimated marginal posterior predictive distributions are illustrated using 99% quantiles (light blue region) as well as mean (black line) and median (blue line). The experimental data (total cell mass) is illustrated using red color

Yeast colonies and biofilms represent an efficient experimental model system to investigate how metabolic dynamics and spatial coupling determine morphogenesis because yeast exhibits (i) cell state transition in dependence on the environment by switching from glucose to ethanol metabolism and quiescence [7], (ii) fast growth, and (iii) can be easily genetically modified (Fig. 1A, B). Although yeast is a unicellular organism, it can form rather complex colony structures including heterogeneous cell states in a strain specific manner [8, 9]. Recently, we have shown that predominant changes in morphology from smooth to wrinkled “fluffy” structures can be induced by aneuploidy as a multicellular phenotype switch [10]. Despite the systematic genetical characterization of this switch, the question how the gain or loss of a chromosome copy leads to a significant change in morphology is not understood.

Mathematical modeling can provide essential insights into the underlying processes as it allows quantitative investigation of the coupling between metabolic and spatial growth dynamics. A general challenge is thereby to cover and parameterize the relevant scales ranging from intra- and intercellular interactions to population and environment dynamics. Existing multiscale modeling approaches for complex multicellular systems typically rely on large sets of physiological parameters that are often not easily accessible in experiments [11, 12]. Other spatiotemporal modeling approaches are based on homogeneity assumption and simulate partial differential equations neglecting the discrete properties of cells. While being useful in building a general understanding of different mechanisms across the scales, most of these approaches do not allow for direct experimentally-based model construction and validation. Such experimental data driven model constructions have been successfully applied in the context of mechanistic modeling of molecular mechanisms [13–15] and extending these approaches to more complex multiscale models will be essential for methodological advancement in systems biology [16].

Here, we develop such a new multiscale modeling framework for multicellular yeast structure formation that allows for experimentally-based model construction and validation. In contrast to previous approaches that simulate individual cells [17], our framework is based on an approximation that discretizes the spatial domain into elementary cubes and allows us to model the heterogeneous microenvironment dynamics under the local homogeneity assumption. Furthermore, the elementary cube approximation enables us to model the information flows (like nutrient transport or the flow of signaling molecules) and mass transfer (movement of the growing cell mass) by means of computationally efficient flux mechanisms. The presented model represents a first approach to simulate colony growth in a data driven manner but does not address aneuploidy particularly as the underlying mechanism at this stage.

To construct a growth and cell state model for the homogeneous microenvironment dynamics, we combine ordinary differential equation (ODE) modeling with experimental data using advanced statistical techniques and, by means of this objective approach, infer the metabolic switching mechanisms as well as the corresponding model parameterization directly from the data. The calibrated microenvironment model is subsequently embedded into the spatial framework which allows for predictions of cell mass, cell state, nutrient, and metabolic distributions throughout the colony formation process after model calibration by colony growth data.

Our model construction process utilizes measurements from two different yeast strains. First, we calibrate the model using time-course data from wild-type yeast cells (YAD145) and subsequently the calibrated model is validated against independent measurements from a respiratory deficient (petite) yeast strain (YAD479). These genotypically different training and validation strains are known to result in distinct colony morphologies and therefore the validation approves that our multiscale model captures essential mechanisms across the scales spanning from microenvironment dynamics to the spatiotemporal colony formation dynamics.

## Results

### Dynamic model construction for cell growth and metabolic switching in homogeneous medium

Depending on external conditions and their intracellular state, yeast cells can either metabolize glucose or ethanol for growth or remain in the so-called quiescent state. The diauxic shift between the different metabolic states is determined by nutrient sensing pathways and if the extracellular glucose level becomes low, cells change their metabolic wiring towards a state that allows growth on ethanol produced during growth on glucose [7, 18]. Cells can also switch to a quiescent state in which they act as passive by-standers that do not grow nor produce any aromatic alcohols. The metabolically distinct glucose, ethanol, and quiescent cell states are the starting point in our model construction and a schematic illustration of the dynamic interactions between these states is shown in Fig. 1B.

The dynamics of the different cellular metabolic states cannot be easily observed directly but it is rather straightforward to monitor cell growth by optic growth curve measurements [19] (see “Methods” section). With the help of mathematical modeling, we are able to infer the switching behavior between the metabolic states and the related nutrient dynamics from time-course data. This is done by constructing alternative quantitative growth models with different metabolic switching mechanisms between the states and testing these hypothetical models against time-course data by means of statistical techniques. In the following, we construct a mathematical model that describes yeast cell growth on glucose and ethanol and couples the growth dynamics with transient switching between three distinct metabolic states: (i) glucose, (ii) ethanol, and (iii) quiescent state (Fig. 1B).

We model the cell growth and switching between different metabolic states by ODEs. We start by considering the glucose state in which the cells grow on glucose and denote the cell mass in this state by *m*^g^. Given that glucose intake is sufficiently fast, the cell mass dynamics in the glucose state can be modeled as

1 $$ \frac{dm^{\mathrm{g}} }{d t} = \mu_{1} m^{\mathrm{g}} g - \beta_{1} \frac{1}{g + K} m^{\mathrm{g}} - \beta_{2} m^{\mathrm{g}},  $$

where *g* denotes the level of available glucose and the first term, *μ*_1_*m*^g^*g*, describes the actual growth kinetics with the rate parameter *μ*_1_. If the glucose signal drops to a low level, cells start to switch gradually to the ethanol state. This switching is reflected by the second term in Eq. 1 with the switching rates *β*_1_ and *K*. Analogously, the third term in Eq. 1 describes the potential switching to the quiescent state with the rate parameter *β*_2_. In a typical experimental setting, a fixed amount of glucose is provided to cells in the beginning and the glucose level decreases when it is used for growth. Subsequently, the glucose concentration is governed by

2 $$ \frac{dg }{d t} = - \frac{\mu_{1}}{\gamma_{1}} m^{\mathrm{g}} g,  $$

where *γ*_1_ is a parameter that determines the yield of glucose to the produced biomass. Growth in the ethanol state occurs in an analogous manner as in the glucose state. We denote the cell mass in the ethanol state by *m*^e^ and the cell mass dynamics in this state is modeled as

3 $$ \frac{dm^{\mathrm{e}}}{d t} = \mu_{2} m^{\mathrm{e}} e + \beta_{1} \frac{1}{g + K} m^{\mathrm{g}} -\beta_{3}m^{\mathrm{e}}.  $$

Here, the first term describes the actual growth kinetics with the rate parameter *μ*_2_, the second term corresponds to the cell mass entering the ethanol state from the glucose state, and the third term describes the possible switching from the ethanol state to the quiescent state with the rate parameter *β*_3_. Ethanol is typically not added to a cell culture, but it is produced as a by-product of growth on glucose. Thus, the ethanol dynamics is given by

4 $$ \frac{de }{d t} = \frac{\mu_{1}}{\gamma_{2}} m^{\mathrm{g}} g - \frac{\mu_{2}}{\gamma_{3}}m^{\mathrm{e}} e,  $$

where the first term represents ethanol production during the growth on glucose and the second term considers the decrease due to biomass production. The parameters *γ*_2_ and *γ*_3_ determine the production and decrease, respectively. The above expressions for *m*^g^ and *m*^e^ dynamics include switching to a quiescent state. We denote the cell mass in the quiescent state by *m*^q^ and describe the cell mass dynamics in this state by

5 $$ \frac{dm^{\mathrm{q}} }{d t} = \beta_{2} m^{\mathrm{g}} + \beta_{3} m^{\mathrm{e}},  $$

with the terms introduced in Eqs. 1 and 3. Given the three distinct metabolic states, the total cell mass reflecting directly the experimental time-course measurements is given by *m*=*m*^g^+*m*^e^+*m*^q^. In experiments, cells are initially put in glucose rich medium and we therefore assume that all cells are initially in the glucose state and the initial glucose level is high. Consequently, we assume that only the model variables *m*^g^ and *g* have non-vanishing initial values. These properties are also used in the reparameterization of the mathematical model which is presented in detail in Additional file 1. The model output, i.e. the total cell mass as a function of time, is denoted by *m*(*t*,*θ*) where *θ* is a parameter vector containing the parameters that result from the reparameterization.

### Statistical inference for model parameters and metabolic transitions in homogeneous medium

The mechanisms that are included in the mathematical model are illustrated in Fig. 1B. The full model contains the essential transition from the glucose state to the ethanol state and allows the cells also to switch to the quiescent state directly from the glucose and ethanol states. However, detailed information about the switching mechanisms to the quiescent state is not available and, consequently, there remains notable uncertainty about the routes that cells may use to enter the quiescent state. To treat this uncertainty accurately, we consider three alternative hypotheses (*H*_1_,*H*_2_, and *H*_3_) regarding the switching routes between the metabolic states (schematic illustrations of corresponding switching models are shown in Fig. 1C) and investigate the feasibility of these hypotheses by quantitative statistical testing. In the following, we outline the experimental data used for model calibration and explain how we infer the structure and parameterization of the microenvironment model.

To obtain dynamic data on total cell mass that can be used in the microenvironment model inference, we measured growth curves for wild-type and petite yeast strains (see “Methods” section). The petite yeast strain differs genetically from the wild-type strain and is not capable to grow on ethanol [10, 20]. In the context of our microenvironment model, this means that the growth rate parameter *μ*_2_ should tend to zero when the petite strain is considered but all other parameters can be expected to be shared between these two strains. Given this direct connection between the wild-type and petite strains, we can carry out the statistical inference using the wild-type data and subsequently test the predictive performance of our models against the petite strain which is not included in the model calibration.

For model inference, we first collect the wild-type growth curve data into the data vector *D*_*k*_. The elements of this data vector contain the average total cell mass at time points *t*_*k*_,*k*=1,…,*N*. The average cell mass as well as the corresponding sample variances *v*_*k*_ are computed over 6 replicates (see Additional file 1: Figure S1 for details about data pre-processing). From previous studies [5, 18, 21] the relative fractions of cells in ethanol and quiescent states at steady state (reached in our setting at *t*_*N*_=80 hours) can be taken to be approximately 29±6*%* and 62±6*%*, respectively. We denote these relative fractions by *α*^e^=0.29 and *α*^q^=0.62 and the corresponding standard deviations representing uncertainty about the exact values by $\phantom {\dot {i}\!}\sigma _{\alpha ^{\mathrm {e}}}= 0.02$ and $\phantom {\dot {i}\!}\sigma _{\alpha ^{\mathrm {q}}}=0.02$. These wild-type data, which are used in the model calibration and hypothesis testing, can be combined with the model output under alternative metabolic switching hypothesis *H*_1_,*H*_2_, and *H*_3_ by assuming independent normally distributed measurement errors and defining the likelihood function

6 $$\begin{array}{*{20}l} \pi(D|\theta_{H_{i}},H_{i}) = \prod_{k = 1}^{N} &\mathcal{N}\left(D_{k} | m_{H_{i}}(t_{k},\theta_{H_{i}}), v_{k}\right) \\ \times & \mathcal{N} \left(\alpha^{\mathrm{e}} \left| \frac{m_{H_{i}}^{\mathrm{e}}(t_{N},\theta_{H_{i}})}{m_{H_{i}}(t_{N},\theta_{H_{i}})}\right., \sigma^{2}_{\alpha^{\mathrm{e}}}\right)\\ \times\quad&\mathcal{N} \left(\alpha^{\mathrm{q}} \left| \frac{m_{H_{i}}^{\mathrm{q}}(t_{N},\theta_{H_{i}})}{m_{H_{i}}(t_{N},\theta_{H_{i}})}\right., \sigma^{2}_{\alpha^{\mathrm{q}}}\right), \end{array} $$

where $D = \left \{D_{k},v_{k},\alpha ^{\mathrm {e}},\sigma _{\alpha ^{\mathrm {e}}},\alpha ^{\mathrm {q}}\sigma _{\alpha ^{\mathrm {q}}}\right \}$ is the data, $\theta _{H_{i}}$ is the parameter vector under the hypothesis *H*_*i*_, and $\mathcal {N}\left (\cdot | \mu, \sigma ^{2}\right)$ is the normal probability density function with the mean *μ* and variance *σ*^2^. We next construct a Bayesian statistical model by combining the likelihood function with uninformative but proper prior distributions where we do not assume any prior dependencies between the parameters and use standard normal prior distributions in logarithmic parameter space. The selected prior distribution introduces a soft lower bound for the parameters. Thus, if a certain rate parameter is present in the model, its value cannot be infinitely close to zero. We estimate the parameter posterior distributions and posterior probabilities of alternative hypotheses by means of population-based Markov chain Monte Carlo (MCMC) sampling and thermodynamic integration (see “Methods” section for details).

### Quantitative hypothesis testing reveals the most likely metabolic switching mechanisms

The posterior analysis is first carried out independently for each alternative metabolic switching mechanism (hypotheses *H*_1_,*H*_2_, and *H*_3_). The resulting approximations for the parameter posterior distributions show that the models are identifiable under all three metabolic wiring scenarios (Additional file 1: Figures S2-S4 and a summary about convergence diagnostics in Figure S5). In general, the predictions in all three scenarios are in a good agreement with the experimental wild-type data (see predicted total cell mass in Fig. 1C, wild type). The posterior predictive distributions (PPDs) are very similar under the hypotheses *H*_1_ and *H*_2_ and the only notable difference is a larger dynamical variability under *H*_1_ (Fig. 1C, Wild type). This finding is consistent since the models are nested and the additional switching route under hypothesis *H*_1_ increases the model flexibility. The PPD under hypothesis *H*_3_ exhibits less variability and additionally a distinct dynamic behavior of *m*^e^ compared to the other two scenarios. Furthermore, Fig. 1C shows the PPDs also for the petite strain and we can conclude that under all three hypotheses we are capable of predicting the total cell mass dynamics of the petite strain even though the dynamics of the non-observed model components may differ significantly. Consequently, we can conclude that the predictive performance of our models is good for both the training and the validation data sets. However, based on visual inspection, it is impossible to judge which hypothesis is most likely and, therefore, we perform statistically rigorous quantitative hypothesis testing over the hypotheses *H*_1_,*H*_2_, and *H*_3_.

Despite the non-distinguishable model predictions in the data space, the posterior analysis over different metabolic switching hypotheses shows significantly more evidence for *H*_2_ (Fig. 1C) with a posterior probability of *H*_2_ very close to 1 (the posterior probabilities as well as the estimated logarithmic marginal likelihoods are shown in parentheses after the hypothesis labels in Fig. 1C). This strong statistical evidence for *H*_2_ suggests that the metabolic switching to the quiescent state in wild-type yeast cells occurs always through the ethanol state in agreement with the current biological interpretations [7, 18, 22].

### Spatial modeling framework to study colony formation

In our experimental setup, yeast cells grow on a glucose rich agar plate and form 3d colonies (Fig. 1A) but the underlying growth mechanisms in terms of metabolic activity and cell state transitions are not understood. To address this challenge, we construct a spatial modeling framework which allows us to predict three dimensional cell state and nutrient distributions during the colony formation process based on our inferred microenvironment model. In addition to cell mass and nutrient dynamics within the colony, we also model the nutrient dynamics within the agar.

**Fig. 2:**
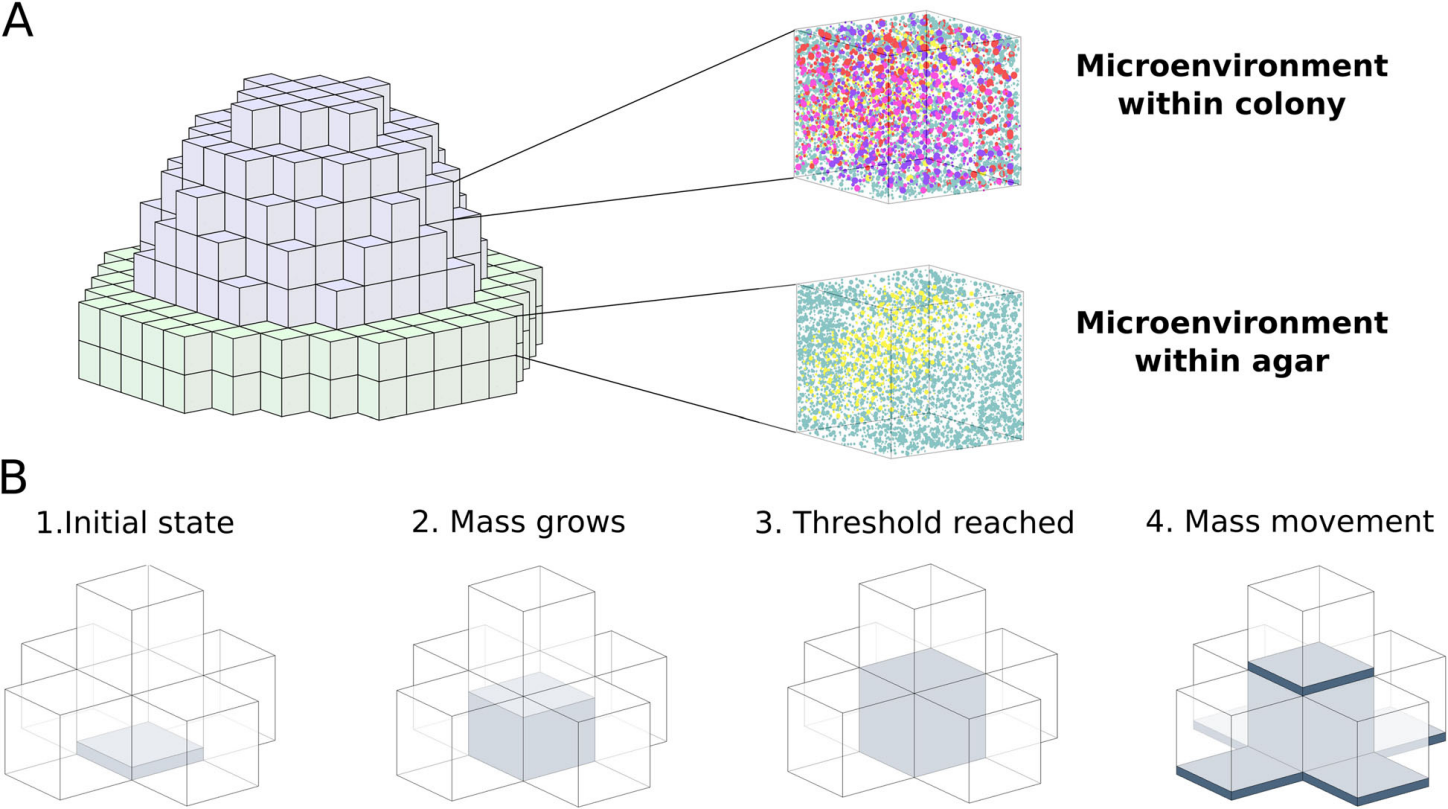
Illustration of the spatial modeling framework. Simulated colonies consist of interacting elementary cubes (for illustrative purposes, the cubes are here notably larger than in practise). **a** Illustration of the elementary cube approximation of a yeast colony. The upper part of the colony (gray elementary cubes) represents the cell mass domain. In these elementary cubes, each microenvironment consists of a mixture of nutrients and cells in different metabolic states. Further, the lower part of the colony (green elementary cubes) represents the nutrient rich agar domain. In the agar domain, each microenvironment can consist of a mixture of nutrients and no cell mass is present. **b** Mass movement is modeled by considering the fill levels of the elementary cubes. The cell mass is growing in the cubes and once a the fill level threshold is reached, cell mass starts to be move into the neighboring cubes. During the cell mass movement, relative fractions of cells in different metabolic states are moved along

To setup the spatial model, we discretize the space into elementary cubes (Fig. 2A). Since the size of the elementary cubes is chosen appropriately, the growth dynamics within each cube (microenvironment) can be modeled under the homogeneity assumption. In other words, each elementary cube consists of a homogeneous mixture of nutrients and cells in distinct metabolic states (Fig. 2A) and the time-evolution of these local components can be described using the microenvironment model developed above. The spatial colony formation is subsequently determined by the dynamics of interacting neighboring cubes with information exchange by the flow of nutrient signals and movement of the growing cell mass.

The cell mass movement is modeled by considering fluxes between neighboring cubes determined by thresholded fill levels of the neighboring cubes where cell mass is moving from a high to low concentration (for illustration see Fig. 2B with parameters given in Table 1). The thresholding is essential because the size of elementary cubes is fixed and it is reasonable to assume that the mass movement does not occur until a certain amount of cell mass has accumulated locally and the resulting pressure starts to push cells forward. In our implementation, the fluxes are computed between six neighboring cubes in each spatial direction and the time-evolution of the full mass distribution is modeled using an ODE system which is determined by the net impact of the individual fluxes. The fluxes are always computed based on the thresholded total mass distribution and the proportions of metabolic states moving along the cell mass are proportional to the proportions of cell states in the cube from which the cell mass is moving. On top of the agar, cell mass can move only to five directions because mass movement into the agar is excluded.

**Table 1:** Parameters of the spatial framework. Bounds are given for parameters that are estimated

	Parameter	Value	Bounds
mass movement threshold	*th*	1	–
mass movement rate	*λ*_mass_	20 h ^−1^	–
nutrient transfer rate within agar	*λ*_agar_	25.42 h ^−1^	[5,75]
nutrient transfer rate within colony	*λ*_col_	0.05 h ^−1^	[0.005,1]
elementary cube edge length	*h*	0.1 mm	–
initial glucose conc. in the agar	$g_{0}^{\text {agar}}$	1	–

The nutrient transfer is modeled using the same flux-based model as the cell mass movement. However, the thresholding is not needed for the nutrient transfer because it can be assumed that nutrients can diffuse freely over the domain. The domain for glucose diffusion is the union of the agar domain and the elementary cubes with positive cell mass. In addition, it is assumed that the ethanol which is produced as a by-product during growth on glucose can diffuse freely over the positive cell mass. A formal derivation of the mass movement and nutrient transfer models can be found in the “Methods” section.

### Data-driven calibration of the spatial model

As explained in detail above, the spatial model consists of interacting elementary cubes and within each cube we consider an approximately homogeneous mixture of cells in different metabolic states and nutrients. Local dynamics in each elementary cube are modeled using the microenvironment model whose structure and parameterization is calibrated using growth curve data and population composition information at 80 hours. More specifically, we use the microenvironment model under metabolic switching hypothesis *H*_2_ which was ranked the highest in the statistical testing. The parameterization of this model is fixed to the maximum a posteriori values that were obtained as a by-product of the posterior analysis. Once the microenvironment model is parameterized, we are left with several unknown parameters that are needed for the spatial framework. These parameters are the mass movement rate, the nutrient transfer rates in the agar and within the cell mass, and the initial glucose level in the agar (Table 1). Because practically no pressure is accumulating inside the colony, we set a high value for the mass movement rate (20 h ^−1^). This means that the cell mass is distributed at the same rate as the cells are growing and local crowding does not occur. Furthermore, we assume that the glucose reserve in the agar can be modeled by means of a disc with thickness of 0.2 mm and a diameter of 1 cm. Then the local initial glucose level in the elementary cubes in the agar domain can be normalized to equal one and, consequently, we are left with two free parameters: the nutrient transfer rate in the agar and the nutrient transfer rate within the cell mass.

To estimate the free parameters of the spatial framework, we measure the colony footprint as the area under the growing wild type colony over time (see “Methods” section for details) and optimize the free parameters by minimizing the difference of the experimental measured footprint and the area under the simulated colony. Hence, we minimize the cost function

7 $$ \xi(\lambda_{\text{agar}},\lambda_{\text{col}}) \,=\, \log\left(\sum_{i = 1}^{n} \left(A_{t_{i}}^{\text{sim}}(\lambda_{\text{agar}},\lambda_{\text{col}}) - A_{t_{i}}^{\text{meas}} \right)^{2} \right),  $$

where *λ*_agar_ and *λ*_col_ are the transfer rates within the agar and the colony, and $A_{t_{i}}^{\text {sim}}(\lambda _{\text {agar}},\lambda _{\text {col}})$ and $A_{t_{i}}^{\text {meas}}$ are the simulated and measured areas at time *t*_*i*_, respectively. Because objective initialization of the cell state and nutrient distribution above the agar is practically impossible, we initialize one elementary cube with cell mass in the glucose state up to the cell mass movement threshold and set the initial glucose level in this cube to one.

**Fig. 3:**
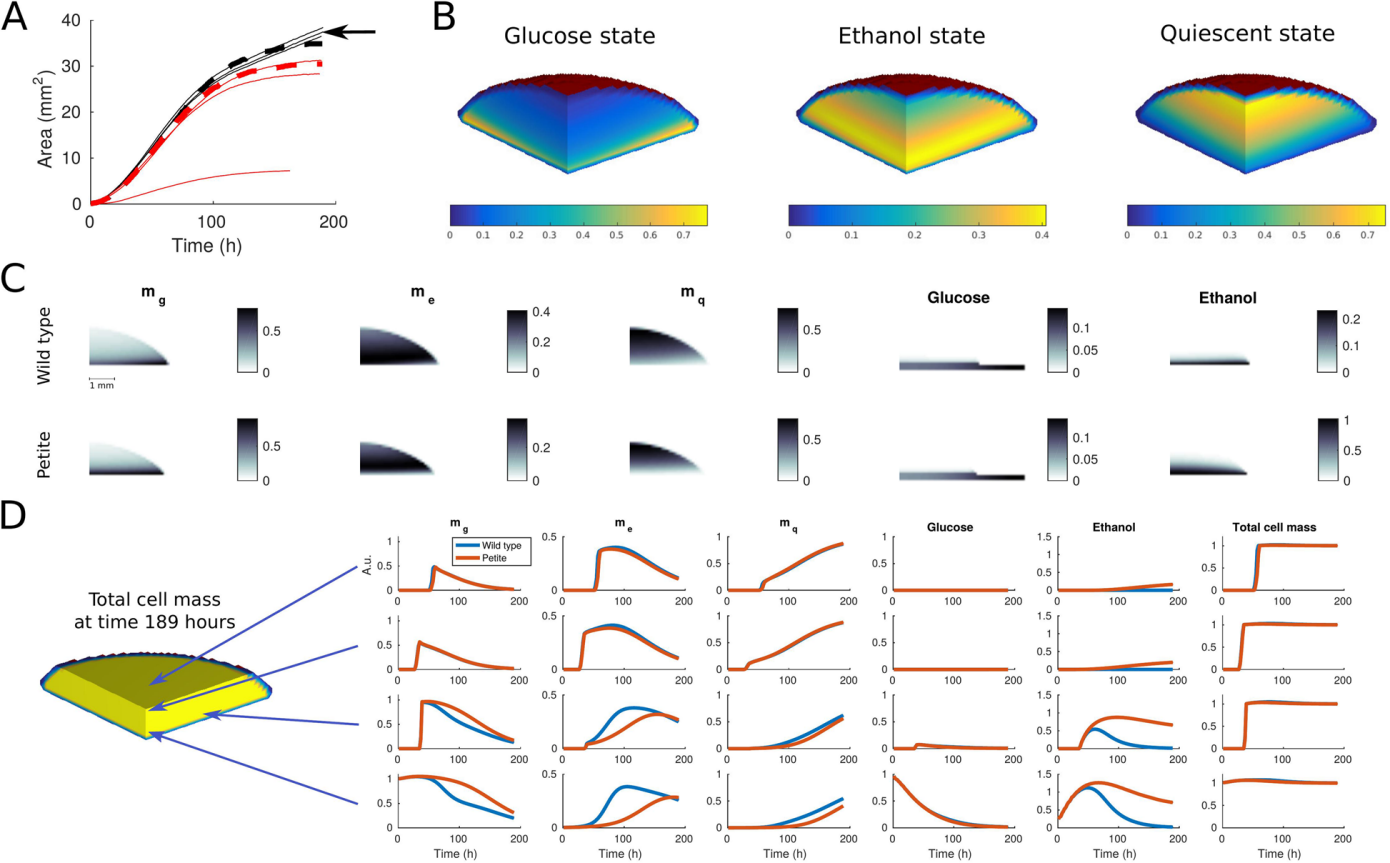
The calibration of the spatial framework and predictions on the colony morphology and colony composition. The colony composition is illustrated for a quarter colony which contains full information of the symmetric colony. **a** Simulated colony footprint areas for wild type and petite strain are plotted using black and red dashed lines, respectively. Experimental data from wild type and petite strains (three replicates from both strains) are plotted using black and red solid lines, respectively. The black arrow indicates the wild type replicate which was used to calibrate the model. The data from the petite strain is used only for validation purposes. **b** Isosurface illustration of the simulated colony shape and cell state composition at time 121 hours. **c** Simulated cell state and nutrient distributions for wild type and petite strains at time 121 hours illustrated using heatmaps. The shown vertical slice is located in the middle of the colony. **d** Simulated time-evolution of all model component all total cell mass at different spatial locations. The exact coornitates (in mm) for illustrated point are (1,1,1),(0.1,0.1,1.0),(0.1,1.5,0.2), and (0.1,0.1,0.1) (starting from the upper row)

We minimize the cost function using Bayesian optimization [23]. The optimization is initialized by evaluating the cost function at 20 points which are sampled within the bounds (Table 1) using Latin hypercube sampling. After initialization, the optimal parameter values (Table 1) are obtained after 9 iterations of the algorithm. Figure 3A exhibits the fitted footprint area against the experimental data. The model fit is in a good agreement with the data even though at the late time points the model shows saturating behavior that is not present in the real data. This slight disagreement suggests that there is some fraction of cells in a metabolic state which is not included in the model. However, the calibrated model does not only fit well to the wild type data but is also in an excellent agreement with two replicates of our petite strain validation data (see red curves in Fig. 3A). The third replicate can clearly be seen as an outlier and may indicate a low efficiency of biomass production [20] described in the model by the yield parameter *γ*_1_. Based on the good fits, we conclude here that our model successfully captures essential dynamics also with respect to the colony size over time.

### Predicting nutrient and metabolic state distributions

The calibrated model provides us with rich information about the spatial organization within the colony as well as the colony morphology over time. Figure 3B illustrates the colony shape and cell state composition at 121 hours. In our data-driven simulation, we observe three distinct regions in which the different cell states are concentrated. The cells in glucose state are present mainly close to the agar, the cells growing on ethanol are located in the middle of the colony, and, in the upper part the colony, we see a high concentration of cells in quiescent state.

A more detailed view on the spatial organization within the colony is given in Fig. 3C which shows the simulated cell state and nutrient distributions for wild type and petite strains in the middle of the colony at time 121 hours. The nutrient distributions show that glucose is mainly present close to the agar and this indicates that most of the glucose growth and consumption occur in this region. Further, the ethanol distributions show that the ethanol level is much higher in case of the petite strain due to lacking consumption. The snapshot distributions for wild type and petite strains look quite similar but essential differences become visible when we observe the time-evolution of model components at different spatial locations (Fig. 3D). Besides the differing ethanol dynamics for wild type and petite strains, also the cell state dynamics differ notably at many spatial locations. The driver for these differences is the growth in the ethanol state which on its behalf affect the switching between the different metabolic states.

## Discussion

We introduced here a novel coarse-grained multiscale model for yeast colony formation demonstrated how computational modeling can be used to integrate experimental information across scales. In particular, we used here growth measurements in homogenous fluid medium to first infer statistically the growth and cell state transition dynamics. For this purpose, we constructed 3 alternative microenvironment models with respect to cell state transitions (Fig. 1C) and implemented rather simple mass action like behavior allowing for efficient model identification. Interestingly, our unbiased approach supports the current biological perspective of the diauxic shift which assumes that cells reach quiescence through the ethanol state [7, 18] by the identification of model *H*_2_.

We subsequently used the microenvironment model in our spatially coarse-grained framework to investigate the impact of metabolic coupling on colony formation. Our coarse-grain approximation can be considered as a compromise between agent based modeling of individual cells typically based on many unknown parameters and continuum strategies by computational expensive partial differential equations. In particular, the coarse-grained cubes allowed the direct incorporation of the inferred microenvironment model and information flux based coupling between neighboring cubes enabled efficient parameter calibration by Bayesian optimization in a data-driven manner. While data-driven model parameterization would be also possible for the other spatial modeling strategies by means of exhaustive parameters sweeps, our approach can save a notable amount of computational resources and increase accuracy by Bayesian optimization. The role of this efficient optimization technique will become even more important when rich data across the scales becomes available and a larger fraction of model parameters can be calibrated together with the spatial parameters.

Here, we applied our modeling strategy to yeast structure formation and could predict how metabolic coupling is shaping the resulting multicellular entity in terms of cell state and nutrient distributions in a dynamical manner (Fig. 3D). Given the available data, our model cannot explicitly discriminate between dead and quiescence cells and therefore does not include growth on dead cell material. As a consequence our predictions do not directly match reported colony organization [24] but correspond to experimental results on yeast biofilm structures where quiescent cells are located at the periphery [9]. This discrepancy indicates the potential consequence of metabolic coupling on multicellular development and distinct regulatory mechanisms. In the current version, our model does also not include the effect of the extracellular matrix (ECM) that can induce intra-colony chambers and channels for nutrient transport to different spatial locations and may support growth under nutrient limited conditions [10]. In this context, our finding on pronounced ethanol and quiescent states within the middle of the colony suggests a preferred interaction of these cells with the ECM and will be investigated in future work. In a similar way, the current version of the model does not include agar invasion of the yeast cells which would induce spatial nutrient gradients leading to structural inhomogeneities. These aspects will be investigated in an extended version of the model by incorporating corresponding data on cell death and agar invasion.

Even though our computational framework is presented in the context of yeast colony modeling, our approach is fully general and can be applied to model any multicellular system. For instance, an interesting future application for our method could be to study the role of metabolic coupling and cellular heterogeneity during human glioblastoma tumor growth [25]. Our ultimate goal is to develop a spatial framework that would allow simultaneous calibration of local and global parameters. Careful formulation of the related statistical inference problem would also enable at least semi-automatic experimental design planning. In other words, the model calibration could be carried out iteratively so that every iteration would not only provide information about the parameters but also probabilistic predictions on the most beneficial future measurements in terms of quantities and time points.

## Conclusions

In this study, we present a data-driven spatial framework to computationally investigate the development of multicellular yeast structure. Throughout the model development process, we used state-of-the-art statistical techniques to handle the uncertainty of model structure and parameterization. Using our unbiased approach, we could validate the underlying mechanism of the diauxic shift and validated the model predictions against independent experimental data illustrating the importance of metabolic coupling in colony formation.

## Methods

### Growth curve data

The experimental procedures are detailed elsewhere [10]. In brief, growth curves in suspension of the FY4 (YAD145) strain and its petite version YAD479 (that is unable to metabolise non-fermentable carbon sources like ethanol) were measured on a TECAN Sunrise (Tecan). Initially, 200 µL were seeded from running cultures at 5×10^5^ cells/mL and cell number was monitored by optical density (OD) every 15 min for 88 hours in YPD medium containing 2% glucose at 30^∘^C. The growth data are provided in a machine readable format as a part of the computational implementation and details about preprocessing can be found in Additional file 1: Figure S1.

### Colony footprint area data

Colony formation was measured by our custom built colony imaging system (Scott and Dudley, unpublished results). Colonies from single cells were grown on YPD-agar plates with 5% glucose for 7.5 days in an incubator at 30^∘^C, and photographed every 20 minutes. Typical distances of colonies at the end point measurements were around 1 cm. Colony areas were extracted from each image by a script for NIH ImageJ [26]. (See Additional file 1 for details on image capture and analysis.)

### Bayesian techniques for ODE model calibration

The parameters and structure of ODE models are calibrated within the Bayesian framework (see e.g. [27]). In brief, we link the model output with time-course data *D* via the likelihood function $\pi (D|\theta _{H_{i}},H_{i})$ where $\theta _{H_{i}}$ is the parameter vector under the hypothesis *H*_*i*_ about the model structure (*i*=1,…,*n*). A Bayesian statistical model can be constructed by combining the likelihood function with a prior distribution over the parameters, $\pi (\theta _{H_{i}}|H_{i})$ [28]. Bayes’ theorem yields the parameter posterior distribution $\pi (\theta _{H_{i}}|D,H_{i}) = \pi (D|\theta _{H_{i}},H_{i})\pi (\theta _{H_{i}}|H_{i})/\pi (D|H_{i})$, where *π*(*D*|*H*_*i*_) is the marginal likelihood. The marginal likelihoods *π*(*D*|*H*_*i*_) can be used to compute the posterior distribution over the hypotheses, i.e. $\pi (H_{i}|D) = \pi (D|H_{i})\pi (H_{i})/\sum _{i}^{n} \pi (D|H_{i})\pi (H_{i})$, where *π*(*M*_*i*_) is the prior distribution over the alternative models. In this study, the prior distribution over the alternative hypotheses is assumed to be uniform.

### Population-based Markov chain Monte Carlo sampling

Neither the posterior distributions nor the marginal likelihoods can be analytically solved for our models and, consequently, the posterior analysis needs to be carried out using numerical techniques. For this purpose, we use the population-based Markov chain Monte Carlo (MCMC) sampling and thermodynamic integration [29, 30].

To implement a population-based Markov chain Monte Carlo sampler, we consider a product form of the target density

8 $$ \pi^{*}\left(\theta_{\beta_{1}},\theta_{\beta_{2}},\dots,\theta_{\beta_{N_{\beta}}}|D,H\right) = \prod_{i = 1}^{N_{\beta}} \pi_{\beta_{i}}\left(\theta_{\beta_{i}}|D,H\right),  $$

where $\pi _{\beta _{i}}(\theta |D,H) \propto \pi (D|\theta H)^{\beta _{i}} \pi (\theta |H)$ is the power posterior for fixed temperatures $0 = \beta _{1} < \dots < \beta _{N_{\beta }} = 1$ [29, 30]. The distributions $\pi _{\beta _{i}}$, including the posterior distribution *π*(*D*|*θ*,*H*)*π*(*θ*|*H*), are marginal distributions of the product form of the target density. By means of population-based MCMC sampling, we draw samples from the individual marginal distributions as well as allow global moves between neighboring temperatures (for details, see [29, 30]).

In this study, we select the temperatures according to the formula

9 $$ \beta_{i} = \left(\frac{i-1}{N_{\beta}-1}\right)^{5}, \quad i = 1,\dots,N_{\beta},  $$

and use altogether 30 temperatures (*N*_*β*_=30). Before running the sampler, we use local gradient-based deterministic multistart optimization to determine the highest peak in each temperature and the corresponding points are then used as an initial state for the sampler. For the multistart optimization, we use our own optimization routine which is implemented in Matlab according to the guidelines given in references [31, 32]. The actual sampling is run in two parts. First, 10^5^ samples are drawn so that the normal proposal distributions are adaptively tuned based on the estimated covariance of the previous 7500 samples. After this burn-in and adaption period, the proposal distributions are fixed and every 1000th sample is collected until 2500 samples are obtained. We run four independent samplers under each alternative hypothesis and the convergence of the chains is monitored by means of the potential scale reduction factors [33] and visual inspection over all temperatures. After checking the convergence, the samples from four independent runs are combined and the posterior analysis is carried out using all 10^4^ samples.

### Bayesian optimization

The parameters of the spatial model are optimized by using the Bayesian optimization technique which is tailored for global optimization of cost functions [23, 34].

To calibrate the spatial model, we need to minimize a target function $y(\mathbf {x}):\mathbb {R}^{d}\rightarrow \mathbb {R}$ with respect to the parameters **x** (we note here that this notation applies only to this subsection). The evaluation of the target function is computationally costly and, to be able to find the minimum using as few as possible function evaluations, we approximate *y*(**x**) by means of a Gaussian process *f*(**x**). Formally, we can write

10 $$ f(\mathbf{x})\sim \mathcal{GP}\left(0,k(\mathbf{x},\mathbf{x}',\boldsymbol{\theta})\right),  $$

where

11 $$ k(\mathbf{x},\mathbf{x}',\boldsymbol{\theta}) = \theta_{d + 1}\exp\left(-\sum_{k = 1}^{d} \frac{(x_{k} - x_{k}')^{2}}{2\theta_{k}^{2}}\right)  $$

is the squared exponential kernel function and $\boldsymbol {\theta } \in \mathbb {R}^{d+1}$ is a parameter vector (for details about Gaussian processes, see e.g. [35]). We assume that the approximation error is normally distributed i.e.

12 $$ y(\mathbf{x}) = f(\mathbf{x}) + \epsilon, \quad\epsilon\sim \mathcal{N}\left(0,\sigma^{2}_{\text{error}}\right).  $$

Based on the above definitions, the prior distribution for the approximated function values *f*_*n*_=*f*(**x**_*n*_),*n*=1,…,*N* is the zero-mean multivariate normal distribution, i.e.

13 $$ \mathbf{f} |\mathbf{X} \sim \mathcal{N}(\mathbf{0},\Sigma_{\mathbf{X},\mathbf{X} }),  $$

where **f**=[*f*(**x**_1_),*f*(**x**_2_),…,*f*(**x**_*N*_)]^′^,**X**=[**x**_1_,**x**_2_,…,**x**_*n*_], and {*Σ*_**X**,**X**_}_*ij*_=*k*(**x**_*i*_,**x**_*j*_,***θ***),*i*,*j*=1,…,*N*. It follows also that

14 $$ \mathbf{y} |\mathbf{X} \sim \mathcal{N}\left(\mathbf{0},\Sigma_{\mathbf{X},\mathbf{X}} + \sigma^{2}_{\text{error}}\mathbf{I}\right),  $$

where we have used the above notation, **y**=[*y*(**x**_1_),*y*(**x**_2_),…,*y*(**x**_*N*_)]^′^, and **I** is the identity matrix. The marginal likelihood is $p\left (\mathbf {y} |\mathbf {X},\boldsymbol {\theta },\sigma ^{2}_{\text {error}}\right)$ where we have explicitly added the kernel parameters ***θ*** and error variance $\sigma ^{2}_{\text {error}}$ to emphasize that the distribution and the marginal likelihood depend on this parameterization.

Given a set of evaluated function values at certain points given by **y**=[*y*(**x**_1_),*y*(**x**_2_),…,*y*(**x**_*N*_)]^′^, we can generate a probabilistic prediction on the function value *y*(**x**^∗^) at an arbitrary point **x**^∗^ in the domain. The prediction about the function value *y*(**x**^∗^) can be generated in form of a random variable *y*^∗^ which follows the joint distribution in Eq. 14. By conditioning *y*^∗^ on the evaluated values, we obtain

15 $$\begin{array}{*{20}l} {} y^{*}|\mathbf{X},\mathbf{y},\mathbf{x}^{*} \sim \mathcal{N}(&\Sigma_{\mathbf{x}^{*},\mathbf{X}}\left(\Sigma_{\mathbf{X},\mathbf{X}} + \sigma^{2}_{\text{error}}\mathbf{I}\right)^{-1}\mathbf{y},\\  (\Sigma_{\mathbf{x}^{*},\mathbf{x}^{*}} & \,+\, \sigma^{2}_{\text{error}}) \,-\, \Sigma_{\mathbf{x}^{*},\mathbf{X}}(\Sigma_{\mathbf{X},\mathbf{X}} \,+\, \sigma^{2}_{\text{error}}\mathbf{I})^{-1} \Sigma_{\mathbf{X},\mathbf{x}^{*}}), \end{array} $$

where $\Sigma _{\mathbf {x}^{*},\mathbf {X}} = \left [k(\mathbf {x}^{*},\mathbf {x}_{1},\boldsymbol {\theta }),k(\mathbf {x}^{*},\mathbf {x}_{2},\boldsymbol {\theta }),\dots,k(\mathbf {x}^{*},\mathbf {x}_{N},\boldsymbol {\theta })\right ], \Sigma _{\mathbf {X},\mathbf {x}^{*}} = \Sigma _{\mathbf {x}^{*},\mathbf {X}}'$, and $\Sigma _{\mathbf {x}^{*},\mathbf {x}^{*}} = k(\mathbf {x}^{*},\mathbf {x}^{*},\boldsymbol {\theta })$. The probabilistic nature of the prediction makes it also possible to predict the next point at which it is most beneficial to evaluate the function value in the context of a minimization problem [23]. The optimal evaluation point can be chosen by finding the point **x**^∗^ which maximizes the expected improvement function

16 $$ \mathrm{E}\left[I(\mathbf{x}^{*})\right] = \mathrm{E}\left[\max(y_{\text{min}} - Y,0)\right],  $$

where *y*_min_ is the minimum of the so far evaluated function values and *Y*=*y*^∗^|**X**,**y**,**x**^∗^ (see e.g. [23] for details and illustrative examples). The expected improvement (Eq. 16) can be expressed in the closed form

17 $$ \mathrm{E}\left[I(\mathbf{x}^{*})\right] = (y_{\text{min}} - \hat{y})\Phi\left(\frac{y_{\text{min}} - \hat{y}}{s} \right) + s\phi\left(\frac{y_{\text{min}} - \hat{y}}{s}\right),  $$

where *ϕ* and *Φ* are the standard normal density and distribution function, respectively, and $\hat {y}$ and *s* are the mean and standard deviation of the normal distribution in Eq. 15, respectively [23].

The actual optimization routine consists of two steps: (i) fitting the response surface by maximizing *p*(**y**|**X**) (Eq. 14) with respect to the hyperparameters $(\boldsymbol {\theta },\sigma ^{2}_{\text {error}})$ and (ii) finding the optimal point for next function evaluation by maximizing the expected improvement (Eq. 16). The steps are carried out sequentially and the response surface is always fitted using a set of evaluated function values which are standardized to have a zero mean and standard deviation of one. In our implementation, the hyperparameters of the Gaussian process model and the next evaluation point with respect to the expected improvement are optimized using fminunc and fmincon optimization routines in Matlab, respectively. The hyperparameter optimization is initialized using parameter values *θ*_1_=*θ*_2_=*θ*_3_=1,*σ*_error_=0.1 which correspond to a smooth Gaussian process response surface. In the context of expected improvement optimization, we utilize a multistart optimization strategy for which the initial points are obtained by means of Latin hypercube sampling (lhsdesign function in Matlab). The sequential procedure is repeated until the expected improvement goes under a threshold (10^−46^ in this study) or the maximum number of iteration steps (i) and (ii) is reached.

### Formal definition of the spatial framework

We discretize the space by dividing it into finite size elementary cubes each having a constant volume (see Fig. 2 for illustration). The cubes are indexed by their location in a 3D array i.e. mass in different metabolic states at different spatial locations can be expressed by writing

$$ m^{\{n\}}_{i,j,k},\quad i = 1,\dots,N_{i},\quad j = 1,\dots,N_{j},\quad k = 1,\dots,N_{k},   $$

where {*n*}∈{g,e,q} denotes the metabolic state. The total mass at each location can be computed by summing the cell masses in distinct metabolic states, i.e.

$$ m_{i,j,k} = m_{i,j,k}^{\mathrm{g}} + m_{i,j,k}^{\mathrm{e}} + m_{i,j,k}^{\mathrm{q}}.   $$

The cubes interact through their fill levels and the cell mass is flowing from a high concentration to a low concentration once a certain threshold is exceeded. The amount of mass exceeding the threshold can be interpreted as pressure that pushes the cell mass onwards. This pressure is computed based on a thresholded total mass distribution over the space. The thresholded total mass at a certain spatial location is defined by

$$ m^{th}_{i,j,k} = \max(m_{i,j,k} - th,0),   $$

where *th* is the threshold parameter.

#### Mass movement

For mass movement modeling, the moving cell mass has to reflect the fractions of cells in different metabolic states. The fractions carried along can be taken to be proportional to the cell state fractions in the source cubes (the cubes from which the mass is moved). Consequently, the mass movement is modled by

18 $$\begin{array}{@{}rcl@{}} \frac{d m_{i,j,k}^{\{n\}}}{d t} &=& \lambda_{m} \left[ F(m_{i,j,k},m_{i-1,j,k},m^{\{n\}}_{i-1,j,k},m^{\{n\}}_{i,j,k})\right.\\ & &+ F(m_{i,j,k},m_{i+1,j,k},m^{\{n\}}_{i+1,j,k},m^{\{n\}}_{i,j,k})\\ & &+ F(m_{i,j,k},m_{i,j-1,k},m^{\{n\}}_{i,j-1,k},m^{\{n\}}_{i,j,k})\\ & &+ F(m_{i,j,k},m_{i,j+1,k},m^{\{n\}}_{i,j+1,k},m^{\{n\}}_{i,j,k})\\ & &+ F(m_{i,j,k},m_{i,j,k-1},m^{\{n\}}_{i,j,k-1},m^{\{n\}}_{i,j,k})\\ & &\left.+ F(m_{i,j,k},m_{i,j,k+1},m^{\{n\}}_{i,j,k+1},m^{\{n\}}_{i,j,k}) \right], \end{array} $$

where *λ*_*m*_ is the mass movement rate parameter,

19 $$\begin{array}{*{20}l} F(m,m^{\prime},m^{\{n\}},m^{\prime\{n\}}) = \\ \left\{ \begin{array}{rl} 0, g(m) = g(m^{\prime})\\ (g(m^{\prime}) - g(m))\frac{m^{\{n\}}}{m},& g(m) > g(m^{\prime})\\ (g(m^{\prime}) - g(m))\frac{m^{\prime\{n\}}}{m^{\prime}},& g(m) < g(m^{\prime}) \end{array}\right. \end{array} $$

and *g*(*m*)= max(*m*−*th*,0) is a function which takes care of the thresholding with the parameter *th*. At the agar-cell mass interface, the mass movement into the agar is prevented by mapping the corresponding values of the function *F* to zero.

To show that the mass is conserved through the movement, we can consider mass movement between two elementary cubes *m* to *m*^′^. Based on our model structure, we have

20 $$\begin{array}{*{20}l} m & = m^{\mathrm{g}} + m^{\mathrm{e}} + m^{\mathrm{q}} \end{array} $$

21 $$\begin{array}{*{20}l} m^{\prime} & = m^{\prime\mathrm{g}} + m^{\prime\mathrm{e}} + m^{\prime\mathrm{q}} \end{array} $$

and the thresholded total cell masses in these two cubes are

22 $$\begin{array}{*{20}l} m^{\text{th}} & = \max(m - th,0) \end{array} $$

23 $$\begin{array}{*{20}l} m^{\prime\text{th}} & = \max(m^{\prime} - th,0). \end{array} $$

Without losing any generality, we can assume *m*^th^>*m*^′th^. Now

24 $$ \frac{d m^{\{n\}}}{d t} \,=\, \lambda_{m} F(m,m^{\prime},m^{\{n\}},m^{\prime\{n\}}) \,=\, \lambda_{m}\frac{m^{\{n\}}}{m} (m^{\prime\text{th}} - m^{\text{th}})  $$

and

25 $$ {\begin{aligned} \frac{d m^{\prime\{n\}}}{d t} &= \lambda_{m} F\left(m^{\prime},m,m^{\prime\{n\}},m^{\{n\}}\right) \\ &\quad= \lambda_{m}\frac{m^{\{n\}}}{m} \left(m^{\text{th}} - m^{\prime\text{th}}\right). \end{aligned}}  $$

From Eqs. 24 and 25, we can deduce

26 $$ \frac{d m^{\{n\}}}{d t} = -\frac{d m^{\prime\{n\}}}{d t},  $$

which proofs mass conservation during the movement. Since the net mass movement defined in Eq. 18 is a sum of six pairwise movements, the mass is conserved also for the net movement.

#### Nutrient transfer

The nutrient transfer can be described in a similar manner as the mass movement but, in this context, we do not need to threshold the distribution because nutrient diffusion occurs freely in the media. Furthermore, nutrient transfer can be simply defined by fluxes between the neighboring cubes whereas in the context of mass movement we had to take the fractions of different cell types into account. If we consider the nutrient concentrations *n*_*i*,*j*,*k*_,*i*=1,…,*N*_*i*_, *j*=1,…,*N*_*j*_, *k*=1,…,*N*_*k*_, the nutrient transfer can be described by

27 $$\begin{array}{@{}rcl@{}} \frac{d n_{i,j,k}}{d t} &=& f(n_{i,j,k},n_{i-1,j,k},\lambda_{\text{agar}},\lambda_{\text{col}})I(m_{i-1,j,k})\\ & &+ f(n_{i,j,k},n_{i+1,j,k},\lambda_{\text{agar}},\lambda_{\text{col}})I(m_{i+1,j,k})\\ & &+ f(n_{i,j,k},n_{i,j-1,k},\lambda_{\text{agar}},\lambda_{\text{col}})I(m_{i,j-1,k})\\ & &+ f(n_{i,j,k},n_{i,j+1,k},\lambda_{\text{agar}},\lambda_{\text{col}})I(m_{i,j+1,k})\\ & &+ f(n_{i,j,k},n_{i,j,k-1},\lambda_{\text{agar}},\lambda_{\text{col}})I(m_{i,j,k-1})\\ & &+ f(n_{i,j,k},n_{i,j,k+1},\lambda_{\text{agar}},\lambda_{\text{col}})I(m_{i,j,k+1}). \end{array} $$

Here,

28 $$\begin{array}{*{20}l} f(&n_{i,j,k},n^{\prime}_{i^{\prime},j^{\prime},k^{\prime}},\lambda_{\text{agar}},\lambda_{\text{col}}) = \\& \left\{ \begin{array}{rl} \lambda_{\text{col}} (n^{\prime} - n), &\text{if } k > h + 1 \text{ or } k \,=\, h + 1;k'= k + 1, \\ \lambda_{\text{agar}} (n^{\prime} - n), & \text{otherwise} \end{array}\right. \end{array} $$

where *λ*_col_ and *λ*_agar_ are the nutrient transfer rate parameters within the colony and agar, respectively, and *h* is the height of the agar given as the number of elementary cube layers. The domain in which the nutrient transfer occurs is determined by the indicator function

29 $$ I(m) = \left\{ \begin{array}{rl} 1, &\text{if} m > 0 \\ 0, & \text{otherwise}. \end{array}\right.  $$

In other words, the mass distribution dependent domain for the nutrient transfer consists of the cubes which have a positive cell mass concentration.

### Computational implementation

Mathematical models, population-based MCMC sampler, and Bayesian optimization were implemented in Matlab (The MathWorks Inc., Natick, MA, USA). ODE systems were solved using the ode15s solver and the full multiscale model was simulated using the Euler method with a time-step of 0.0025 h.

## Supplementary information

**Additional file 1** The supplementary information consists of three sections: 1. Colony footprint area data, 2. Reparameterization of the microenvironment model, and 3. Supplementary Figures.

## Data Availability

The datasets generated and analyzed during the current study as well as the computational implementation to reproduce the results are available at https://research.cs.aalto.fi/csb/software/.

## Abbreviations

MCMC: Markov chain Monte Carlo
OD: Optical density
ODE: Ordinary differential equation
PPD: Posterior predictive distribution
YPD: Yeast extract peptone dextrose

## References

[1] Ratcliff WC, Denison RF, Borrello M, Travisano M (2012). Experimental evolution of multicellularity. Proc Nat Acad Sci.

[2] Komin N, Skupin A (2017). How to address cellular heterogeneity by distribution biology. Curr Opin Syst Biol.

[3] Stovivcek V (2010). General factors important for the formation of structured biofilm-like yeast colonies. Fungal Genet Biol.

[4] Granek JA (2013). The genetic architecture of biofilm formation in a clinical isolate of saccharomyces cerevisiae. Genetics.

[5] Taylor MB, Ehrenreich IM (2014). Genetic interactions involving five or more genes contribute to a complex trait in yeast. PLOS Genetics.

[6] Zahn LM, Purnell BA (2016). Genes under pressure. Science.

[7] DeRisi JL (1997). Exploring the metabolic and genetic control of gene expression on a genomic scale. Science.

[8] Vachova L (2009). Architecture of developing multicellular yeast colony: spatio-temporal expression of ato1p ammonium exporter. Environ Microbiol.

[9] Vachova L (2011). Flo11p, drug efflux pumps, and the extracellular matrix cooperate to form biofilm yeast colonies. J Cell Biol.

[10] Tan Z (2013). Aneuploidy underlies a multicellular phenotypic switch. Proc Natl Acad Sci USA.

[11] Kang S (2014). Biocellion : accelerating computer simulation of multicellular biological system models. Bioinformatics.

[12] Doloman A (2017). Modeling de novo granulation of anaerobic sludge. BMC Syst Biol.

[13] Schulz EG (2009). Sequential polarization and imprinting of type 1 T helper lymphocytes by interferon-gamma and interleukin-12. Immunity.

[14] Intosalmi J, et al. Analyzing Th17 cell differentiation dynamics using a novel integrative modeling framework for time-course RNA sequencing data. BMC Syst Biol. 2015; 9(81). 10.1186/s12918-015-0223-6 PMC4650136 26578352

[15] Chan YH (2016). A subpopulation model to analyze heterogeneous cell differentiation dynamics. Bioinformatics.

[16] Skupin A (2010). Calcium signals driven by single channel noise. PLoS Comput Biol.

[17] Walther T (2004). Mathematical modeling of regulatory mechanisms in yeast colony development. J Theor Biol.

[18] Galdieri L (2010). Transcriptional regulation in yeast during diauxic shift and stationary phase. OMICS.

[19] Jung PP, Christian N, Kay DP, Skupin A, Linster CL (2015). Protocols and programs for high-throughput growth and aging phenotyping in yeast. PLoS One.

[20] Day M (2013). Yeast petites and small colony variants: for everything there is a season. Adv Appl Microbiol.

[21] Alvarez-Vasquez F (2007). Coordination of the dynamics of yeast sphingolipid metabolism during the diauxic shift. Theor Biol Med Model.

[22] Aragon AD (2008). Characterization of differentiated quiescent and nonquiescent cells in yeast stationary-phase cultures. Mol Biol Cell.

[23] Jones DR (1998). Efficient Global Optimization of Expensive Black-Box Functions. J Global Optim.

[24] Váchová L, Cáp M, Palková Z (2012). Yeast colonies: a model for studies of aging, environmental adaptation, and longevity. Oxidative Med Cell Longevity.

[25] Dirkse A, Golebiewska A, Buder T, Nazarov PV, Muller A, Poovathingal S, Brons NH, Leite S, Sauvageot N, Sarkisjan D (2019). Stem cell-associated heterogeneity in glioblastoma results from intrinsic tumor plasticity shaped by the microenvironment. Nature Commun.

[26] Schneider CA (2012). NIH Image to ImageJ: 25 years of image analysis. Nature Methods.

[27] Girolami M (2008). Bayesian inference for differential equations. Theor Comput Sci.

[28] Robert CP. The Bayesian Choice: From Decision-Theoretic Foundations to Computational Implementation, 2nd: Berlin and Heidelberg; 2007. https://www.springer.com/gp/book/9780387952314.

[29] Jasra A (2007). On population-based simulation for static inference. Stat Comput.

[30] Calderhead B, Girolami M (2009). Estimating Bayes factors via thermodynamic integration and population MCMC. Comput Stat Data An.

[31] Raue A (2013). Lessons learned from quantitative dynamical modeling in systems biology. PLoS ONE.

[32] Raue A (2015). Data2dynamics: a modeling environment tailored to parameter estimation in dynamical systems. Bioinformatics.

[33] Gelman A, et al. Bayesian Data Analysis, 3rd: Boca Raton. Texts in Statistical Science; 2013.

[34] Ghahramani Z (2015). Probabilistic machine learning and artificial intelligence. Nature.

[35] Rasmussen CE, Williams C. Gaussian Processes for Machine Learning: MIT Press; 2006.

