# Supplementary material for: Data-driven multiscale modeling reveals the role of metabolic coupling for the spatio-temporal growth dynamics of yeast colonies
Source: BMC Mol Cell Biol. 2019 Dec 19;20:59. doi: 10.1186/s12860-019-0234-z (PMC6923950; doi:10.1186/s12860-019-0234-z)
Supplement: Supplementary file 1 — Additional file 1 The supplementary information consists of three sections: 1. Colony footprint area data, 2. Reparameterization of the microenvironment model, and 3. Supplementary Figures. [file 12860_2019_234_MOESM1_ESM.pdf]

Supplementary Information

Data-driven multiscale modeling reveals the role of metabolic coupling for the spatio-temporal growth dynamics of yeast colonies

Contents

1 Colony footprint area data **1**

2 Reparameterization of the microenvironment model **2**

3 Supplementary Figures **3**

1 Colony footprint area data

Growth of colonies was captured photographically over time. Initially, 48 cells were seeded on agar plates (OmniTrays from ThermoFisher with 35mL YPD-agar) containing 5% glucose by FACS (Sony SH800) in an 8 × 12 checkerboard pattern as described in (YIMAA Biotechniques REF). Automated image acquisition of colonies growing on these agar plates at 30°C was performed by a PC-controlled conventional digital camera (Canon 5D Mark II) on a motorised stage. The camera was equipped with a Canon MP-E 65mm macro lens. Lighting was provided by two neutral white (4100K) LEDs (Luxeon Star), driven at 700 mA. All images were exposed for 1/6 s at f/13 and ISO 200. Each plate was divided into 16 regions (each containing 3 colonies) and each region was photographed every 20 min over the course of the 180 h time course. Images were captured at 156 px/mm resolution. Images were saved to a file server as JPGs with a filename composed of the name of the plate, the imaging region, the date and time, and a serial number.

Area measurement
Colony areas were extracted using a custom script for ImageJ (the script is available along with the computational implementation). Each agar plate is divided into 16 imaging regions. The script opens the image files for a region of the agar plates, converts the images to 8-bit grayscale, and thresholds the images using ImageJ's built-in "Set Threshold" method. Since the lighting and background intensities were constant across the imaging set, a threshold of intensity 60 (out of a maximum of 255) was used for all images. Each image is divided into 3 sub regions that each contain 1 colony based on the pattern in which colonies were seeded onto the agar, and the area of the segmented colony is measured using ImageJ's built-in "Analyze particles" function. Any objects with an area less than 1000 px^2 or circularity less than 0.5 are excluded. Colony areas are then recorded in a .csv file along with the filename of the image, which includes the image's time stamp. One .csv file is produced for each imaging region, and contains the time course area data for the three colonies located in that imaging region.

2 Reparameterization of the microenvironment model

In the main text, the derivation of the microenvironment model results in the set of ordinary differential equations

$$\frac{dm^{\mathrm{g}}}{dt} = \mu_1 m^{\mathrm{g}} g - \beta_1 \frac{1}{g+K} m^{\mathrm{g}} - \beta_2 m^{\mathrm{g}} \tag{1}$$

$$\frac{dm^{\mathrm{e}}}{dt} = \mu_2 m^{\mathrm{e}} e + \beta_1 \frac{1}{g+K} m^{\mathrm{g}} - \beta_3 m^{\mathrm{e}} \tag{2}$$

$$\frac{dm^{\mathrm{q}}}{dt} = \beta_2 m^{\mathrm{g}} + \beta_3 m^{\mathrm{e}} \tag{3}$$

$$\frac{dg}{dt} = -\frac{\mu_1}{\gamma_1} m^{\mathrm{g}} g \tag{4}$$

$$\frac{de}{dt} = \frac{\mu_1}{\gamma_2} m^{\mathrm{g}} g - \frac{\mu_2}{\gamma_3} m^{\mathrm{e}} e. \tag{5}$$

We know that the initial level of glucose is non-negative and, for the parameter inference purposes, we normalize the corresponding differential equation with respect to the initial glucose level $g(0)$. Formally, this can be expressed by writing

$$\frac{1}{g(0)} \frac{dg}{dt} = -\frac{\mu_1}{g(0)\gamma_1} m^{\mathrm{g}} g. \tag{6}$$

Further, we can reparameterize the above equation and obtain the dimensionless expression

$$\frac{dx_4}{dt} = -\theta_7 x_1 x_4, \tag{7}$$

where $x_1 = m^{\mathrm{g}}$, $x_4 = \frac{g}{g(0)}$, $\theta_7 = \frac{\mu_1}{\gamma_1}$, and the initial condition is $x_4(0) = 1$.

In a similar manner, we can scale the unobserved ethanol dynamics by setting $\gamma_3 = 1$. This results in the expression

$$\frac{de}{dt} = \frac{\mu_1}{\gamma_2} m^{\mathrm{g}} g - \mu_2 m^{\mathrm{e}} e \tag{8}$$

which has the dimensionless form

$$\frac{dx_5}{dt} = \theta_8 x_1 x_4 - \theta_5 x_2 x_5, \tag{9}$$

where $x_5 = e$, $\theta_5 = \mu_2$, and $\theta_8 = \frac{\mu_1}{\gamma_2}$. The full system can then be written in the form

$$\frac{dx_1}{dt} = \theta_1 x_1 x_4 - \theta_2 \frac{1}{x_4+\theta_3} x_1 - \theta_4 x_1 \tag{10}$$

$$\frac{dx_2}{dt} = \theta_5 x_2 x_5 + \theta_2 \frac{1}{x_4+\theta_3} x_1 - \theta_6 x_2 \tag{11}$$

$$\frac{dx_3}{dt} = \theta_4 x_1 + \theta_6 x_2 \tag{12}$$

$$\frac{dx_4}{dt} = -\theta_7 x_1 x_4 \tag{13}$$

$$\frac{dx_5}{dt} = \theta_8 x_1 x_4 - \theta_5 x_2 x_5, \tag{14}$$

where $(x_1, x_2, x_3, x_4, x_5) = (m^{\mathrm{g}}, m^{\mathrm{e}}, m^{\mathrm{q}}, g, e)$, $\theta_2 = \beta_1$, $\theta_3 = K$, $\theta_4 = \beta_2$, and $\theta_6 = \beta_3$. This is the parameterization that we use in model and parameter inference and the model output to be linked with data is $x_1 + x_2 + x_3 = m^{\mathrm{g}} + m^{\mathrm{e}} + m^{\mathrm{q}} = m$.

3 Supplementary Figures

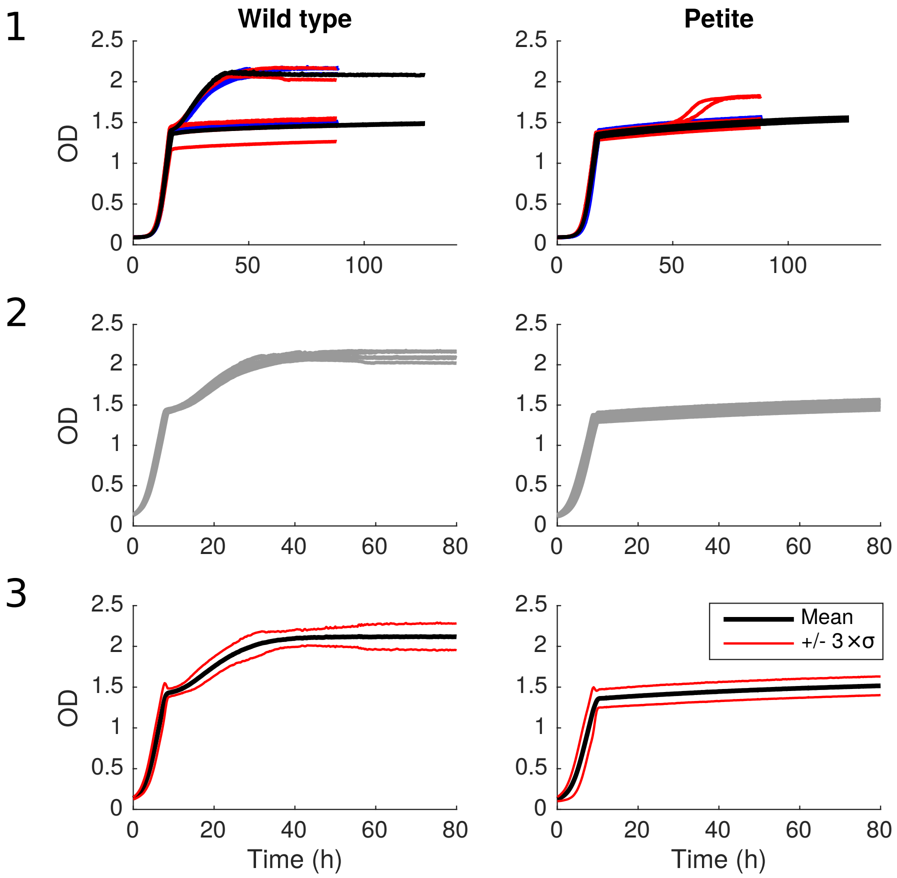

Fig. S1 Three phases of data pre-processing. (1) Raw data is plotted. The color coding indicates from which batch the replicates come from. (2) The outliers are filtered out and the 8 hour lag-phase in the beginning of the measurement is removed. Removal of the lag-phase is needed because we use the data to calibrate the microenvironment model in our spatial framework and, in the spatial context, the cells need to be assumed to have reached the fast growing state when they enter a new microenvironment. (3) The mean and standard deviation is computed.

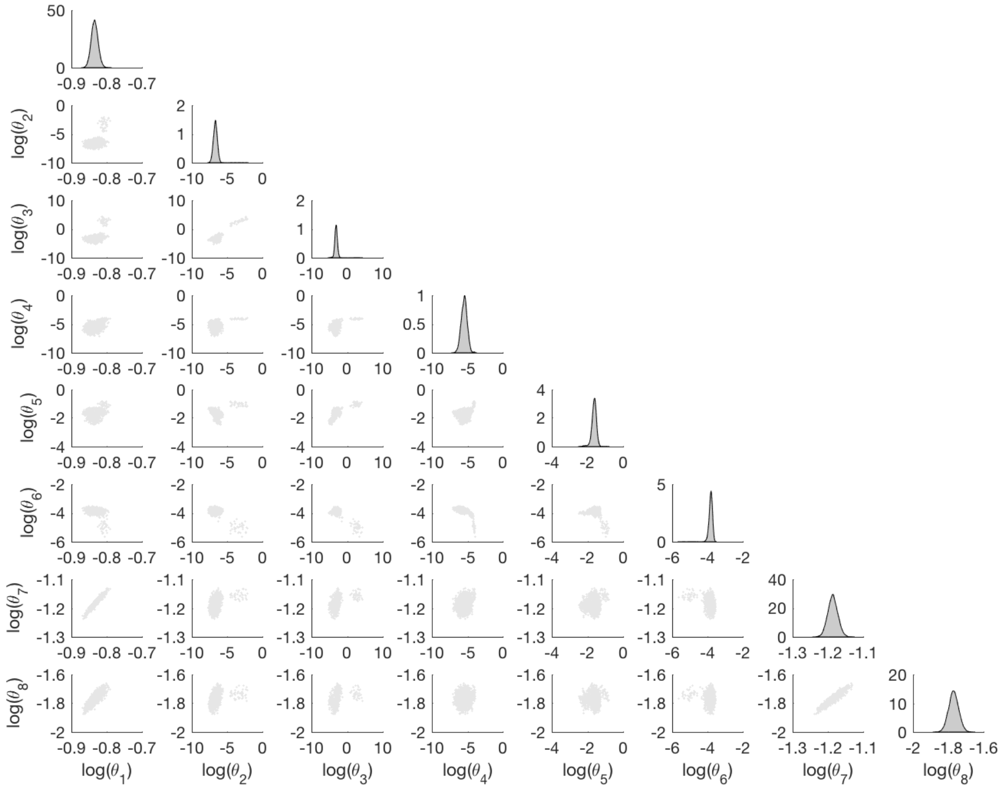

Fig. S2 Illustration of pairwise parameter dependencies and marginal parameter posterior distributions under the hypothesis H_1. The marginal posterior distributions are estimated using a kernel density estimate (ksdensity function in Matlab with default parameters) using all 10^4 samples. The observed slight bimodality is present in all four independent chains and, thus, most likely it is not due to poor convergence.

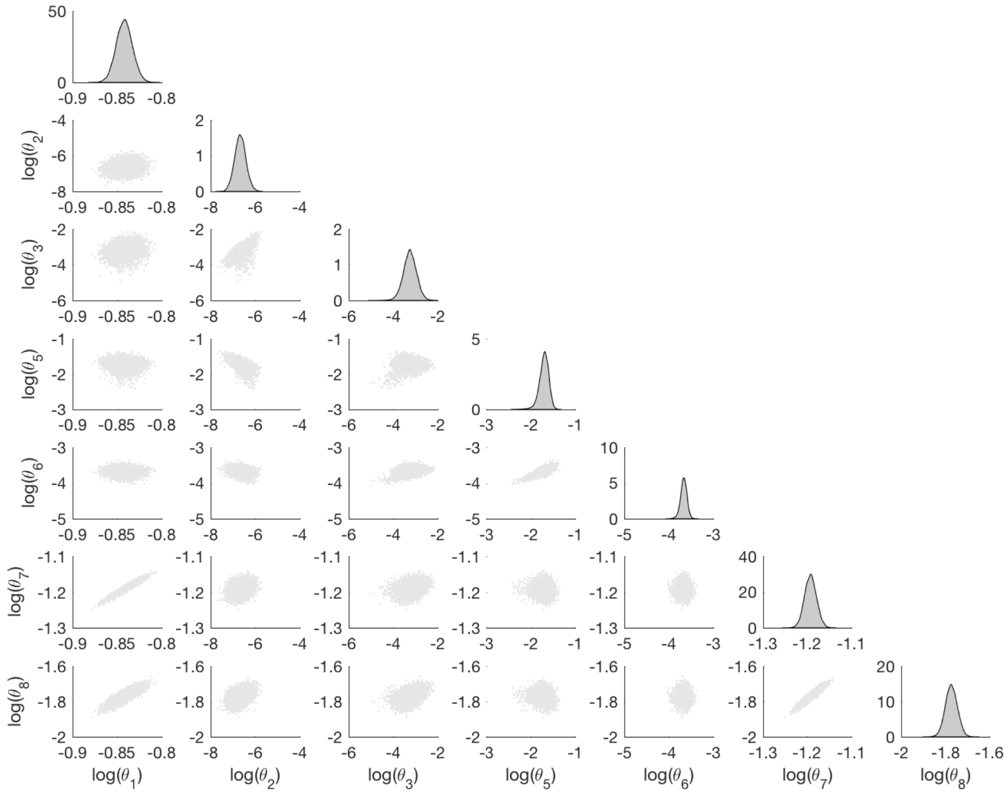

Fig. S3 Illustration of pairwise parameter dependencies and marginal parameter posterior distributions under the hypothesis H_2. The marginal posterior distributions are estimated using a kernel density estimate (ksdensity function in Matlab with default parameters) using all 10^4 samples.

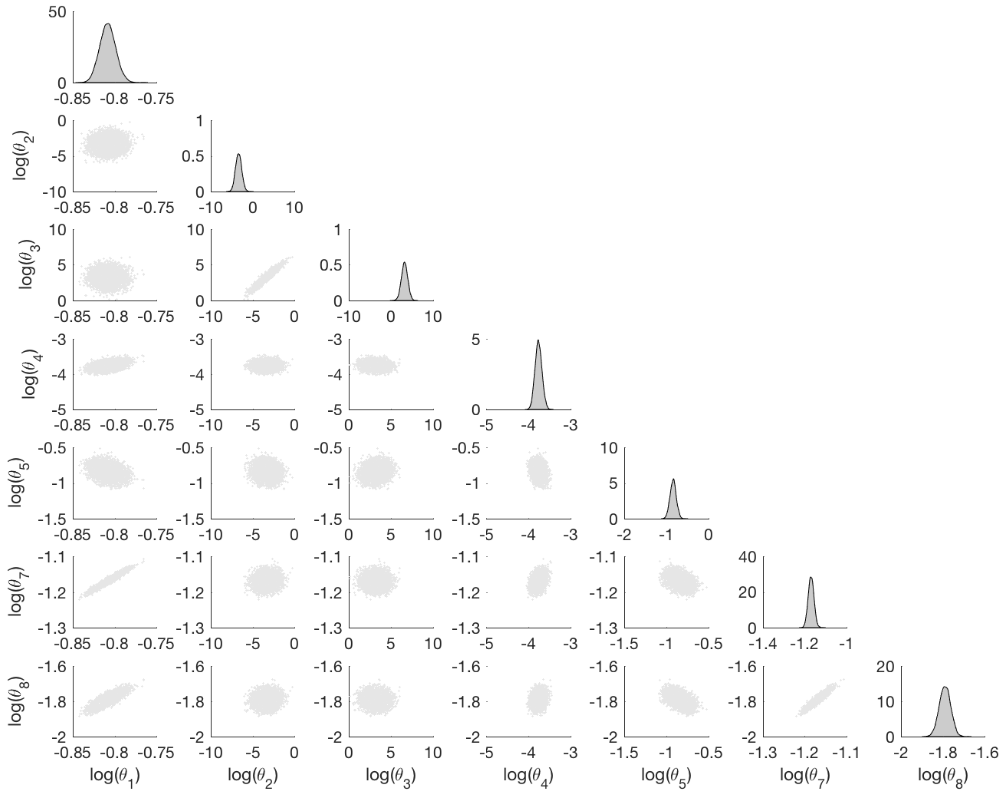

Fig. S4 Illustration of pairwise parameter dependencies and marginal parameter posterior distributions under the hypothesis H_3. The marginal posterior distributions are estimated using a kernel density estimate (ksdensity function in Matlab with default parameters) using all 10^4 samples.

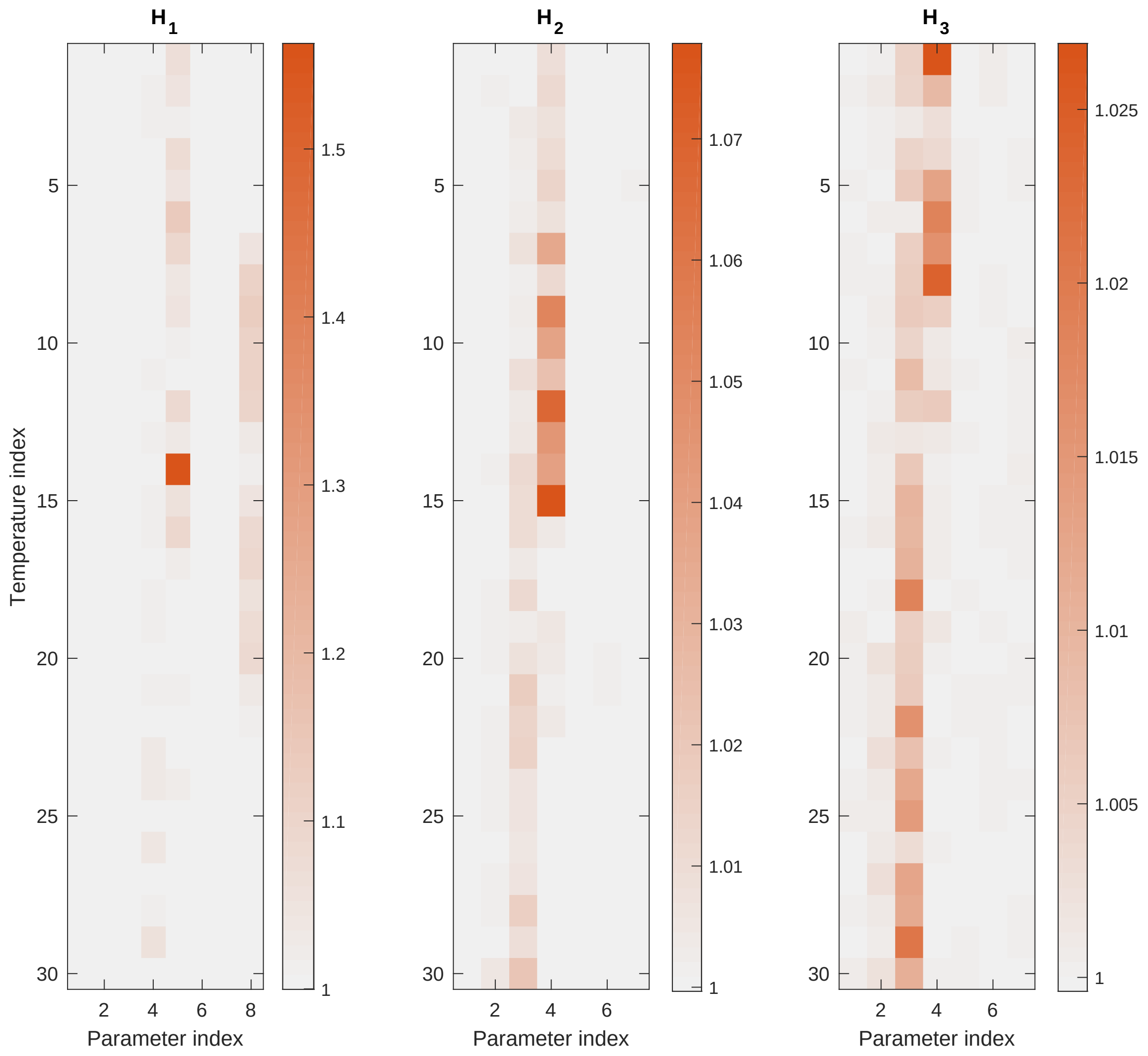

Fig. S5 Potential scale reduction factors (PSRFs) computed using four independent chains under each of the hypotheses H_1, H_2, and H_3. We conclude that, based on the PSRF values and visual inspection of the log-likelihood and sample traces, the chains have converged (see [1] for details about convergence diagnostics).

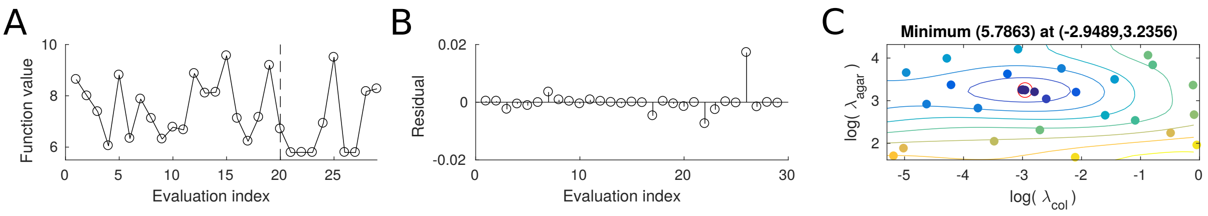

Fig. S6 Summary of the Bayesian optimization run. (A) Values of the cost function at the evaluated points. The first 20 evaluation points are obtained using Latin hypercube sampling and further points (corresponding to the values shown on the right hand side of the dashed line) are selected based on the expected improvement. (B) Residuals computed with respect to the final response surface. (C) The cost function evaluation points are shown as dots, the fitted response surface is illustrated using a contour plot, and the point at which the minimum is reached is indicated using a red circle.

References

[1] A. Gelman *et al.*, *Bayesian Data Analysis.* Chapman & Hall/CRC Texts in Statistical Science, 3rd ed., 2013.
